# Efficacy of insulin in treating severe hypertriglyceridaemia in the third trimester of pregnancy

**DOI:** 10.3389/fmed.2022.977620

**Published:** 2022-11-02

**Authors:** Dong Zhou, Guoqiang Sun, Jing Hu, Quan Gan

**Affiliations:** ^1^Department of Obstetrics, Maternal and Child Health Hospital of Hubei Province, Wuhan, China; ^2^Department of Critical Care Medicine, Maternal and Child Health Hospital of Hubei Province, Wuhan, China

**Keywords:** insulin, third trimester, severe, curative effect, hypertriglyceridaemia

## Abstract

This study aims to investigate the efficacy of insulin in treating severe hypertriglyceridaemia (HTG) during the third trimester of pregnancy. Women with severe HTG (TG ≥ 11.30 mmol/L) in the third trimester of pregnancy who received clinical examination and delivered in Hubei Maternal and Child Health Hospital from 01 January 2017 to 30 September 2021 were recruited. Patients with TG ≥ 11.30 mmol/L at 30–32 weeks of gestation were treated with a low-fat diet and insulin as the insulin treatment group. For the control group, patients with TGs of 5.65–11.30 mmol/L at 30–32 weeks of gestation who developed severe HTG (TG ≥ 11.30 mmol/L) before delivery were treated with a low-fat diet only. General maternal information, delivery, perinatal treatment and laboratory examination information were collected from electronic medical records and compared. We found that in the insulin treatment group, there were higher values of progestational body mass index (BMI) (Z = −2.281, *P* = 0.023), higher incidence of diabetes (χ^2^ = 20.618, *P* < 0.001) and higher incidence of fatty liver (χ^2^ = 4.333, *P* = 0.037) than in the control group but also a higher pregnancy weight gain compliance rate (χ^2^ = 4.061, *P* = 0.044). Laboratory examination before delivery revealed that compared with the control group, insulin treatment significantly decreased prenatal TG (Z = −10.392, *P* < 0.001), cholesterol (Z = −8.494, *P* < 0.001), low-density lipoprotein (Z = −3.918, P < 0.001), apolipoprotein A1 (t = 2.410, *P* = 0.019), cystatin (Z = −4.195, *P* < 0.001), incidence of hypocalcaemia (*P* = 0.036), and absolute number of lymphocytes (Z = −3.426, *P* = 0.001). Delivery outcomes were also improved in the insulin treatment group compared with the control group, including lower neonatal weight (Z = −2.200, *P* = 0.028), incidence of macrosomia (χ^2^ = 4.092, *P* = 0.043), gestational age (Z = −3.427, *P* = 0.001), and rate of intensive care unit (ICU) conversion (*P* = 0.014). In conclusion, insulin therapy for HTG in the third trimester of pregnancy could increase the pregnancy weight gain compliance rate, decrease blood lipid levels and the incidence of severe complications such as HTG acute pancreatitis (HTG-AP), and improve pregnancy outcomes.

## Introduction

Typically, triglycerides (TGs) are mildly elevated during pregnancy and show a gradual decrease to prepregnancy levels after delivery at 6 weeks postpartum. However, hypertriglyceridaemia (HTG) (≥ 5.65 mmol/L) in pregnant women has shown an increasing trend in recent years in China and indicates pregnancy risk. Particularly in the third trimester of pregnancy (>28 weeks), severe HTG (TG ≥ 11.30 mmol/L) can lead to HTG acute pancreatitis (HTG-AP) and other severe complications, such as premature delivery, abortion and even maternal and/or infant death (1). Dietary adjustment is still the basis of lipid management for the treatment of severe HTG in the third trimester of pregnancy (2). The combination of omega-3 fatty acids in the diet could reduce the synthesis of liver TG, increase the activity of lipoprotein lipase (LPL) and safely reduce the TG level of pregnant women by 25–30%. Fenofibrate could be safely used for HTG in the second trimester of pregnancy with slow effects (3). Heparin can reduce serum TG levels, but long-term use of heparin results in reduced chylomicron decomposition, increased TG levels, and rebound HTG as well as potential risk of bleeding. Nicotinic acid was only used in early pregnancy with potential teratogenic effects (4). Blood purification treatment (including haemofiltration, haemoperfusion and plasmapheresis) was safely used to reduce TG levels during pregnancy by replacing deficient LPL or APO when HTG-AP occurred (5). Gene therapy for hyperlipidaemia was reported to reduce the risk of pancreatitis by 70%, but no clinical trials were conducted on pregnant women (6). Insulin was shown to reduce TG by enhancing LPL activity and degradation of chylomicrons. Intensive therapy with insulin during pregnancy was a safe, effective and inexpensive approach for TG reduction, expected to result in reductions of 50–75% within 2–3 days with obvious effects (7). However, there are few clinical studies on the treatment of severe hypertriglyceridaemia with insulin in the third trimester of pregnancy.

Low-fat diet treatment is required for hypertriglyceridaemia (HTG) (TG> 5.65 mmol/L) in pregnant women at 30–32 weeks of gestation in our hospital (The Maternal and Child Health Hospital of Hubei Province). With low-fat diet treatment, only approximately 5% of less severe HTG patients (TG of 5.65–11.30 mmol/L) would become severe HTG (TG ≥ 11.30 mmol/L) before delivery. However, for those with severe HTG (TG ≥ 11.30 mmol/L) at 30–32 weeks of gestation, there is a high risk of developing HTG-AP given low-fat diet treatment alone. Therefore, this study evaluated the efficacy of insulin treatment in patients with severe HTG at 30–32 weeks of gestation compared with a non-insulin-treated patients who had less severe HTG at 30–32 weeks of gestation but developed severe HTG before delivery. We found that patients in the insulin treatment group achieved even better clinical outcomes than those in the control group, although the latter had less severe HTG than the treatment group. Therefore, we concluded that insulin was effective in treating severe hypertriglyceridaemia in the third trimester of pregnancy.

## Materials and methods

### Objects and groups

This study was a retrospective study with parturients who were examined and delivered in the Maternal and Child Health Hospital of Hubei Province from 01 January 2017 to 30 September 2021. All the parturients provided written informed consent for the collection and publication of their medical information during their first visit to the hospital. This study was approved by the Ethics Committee of the Maternal and Child Health Hospital of Hubei Province [Approval Number: [2021] IEC (LW037)].

The objects and groups of this study are shown in the flow diagram (Supplementary Figure 1). Case inclusion criteria: (1) 18–45 years old; (2) examination and delivery in the Maternal and Child Health Hospital of Hubei Province; (3) delivery of a single live birth; (4) HTG in the third trimester (TG> 5.65 mmol/L) (8). Case exclusion criteria: (1) incomplete medical records; (2) non-compliance with medical advice for treatment.

There were a total of 118,622 live parturient women in the Maternal and Child Health Hospital of Hubei Province from 01 January 2017 to 30 September 2021. According to the inclusion and exclusion criteria, there were 48 severe HTG (TG ≥ 11.30 mmol/L) patients who received both a low-fat diet and insulin treatment (insulin treatment group, *n* = 48) and 7,056 with less severe HTG (TG of 5.65–11.30 mmol/L) who only received low-fat diet treatment at 30–32 weeks of gestation. Among the less severe HTG patients, 365 patients developed severe HTG (control group, n=365) before delivery, while the remaining 6,691 patients had non-progressive HTG (i.e., TG remained at 5.65–11.30 mmol/L). Four hundred non-progressive HTG patients were randomly selected for statistical analysis using 6,691 patient IDs as input and the R function “sample” for calculation.

### Therapy

1) Low-fat diet: The main guideline for patients was as follows: Total fat should be limited to 20% of daily calories (9), and foods with a high glycaemic index should be avoided, supplemented with omega-3 fatty acids. The specific type of omega-3 supplements were fish oil soft capsules, and each capsule contained 1000 mg fish oil with 183 mg eicosapentaenoic acid (EPA) and 120 mg docosahexaenoic acid (DHA). The pregnant women took one capsule along with a meal twice a day.
2) Intensive insulin treatment: Insulin was administered when TG ≥ 11.30 mmol/L, with an initial dose of 0.1–0.3 U/kg body weight/hour and continuous intravenous pumping. Blood glucose was monitored once every 1 to 4 h to adjust the pumping dose of insulin. When blood glucose was 8.30–11.10 mmol/L, it was necessary to guard against the occurrence of hypoglycaemia, and 5% glucose (glucose:insulin of (4–6 g):1 U) was to be given at the same time to prevent the occurrence of hypoglycaemia. Insulin pumping was suspended when TG ≤ 5.65 mmol/L. In the event of persistent hypoglycaemia occurs, insulin was to be discontinued (9).
3) Blood purification treatment was guided as follows: If pregnant women have HTG-AP and TG ≥ 11.3 mmol/L at 24–48 h after admission or the decrease in TG does not reach 50% after conservative treatment, blood purification treatment should be adopted, mainly including haemofiltration, haemoperfusion and plasmapheresis (5). For pregnant women without HTG-AP, if the decrease in blood lipids is not significant or the disease progresses and is complicated with hyperlipidaemia pancreatitis, pregnancy is terminated in time and combined with drug therapy and/or blood purification treatment (4).

### Data collection

All information and data were obtained through the hospital information system.

1) General data of puerperas: hospitalization number, age, body mass index (BMI) before pregnancy, weight gain during pregnancy and determination of whether weight gain during pregnancy met the standard were included. A BMI < 18.5 kg/m^2^ was defined as low weight, and the recommended weight gain during pregnancy ranged from 12.5 to 18.0 kg. A BMI of 18.5–24.9 kg/m^2^ was defined as normal body weight, and the recommended weight gain range during pregnancy was 11.5–16.0 kg. A BMI of 25.0–29.9 kg/m^2^ was defined as overweight, and the recommended weight gain range during pregnancy was 7.0–11.5 kg. A BMI ≥ 30.0 kg/m^2^ was defined as obesity, and the recommended weight gain during pregnancy ranges from 5.0 to 9.0 kg (10). The number of deliveries and complications, such as gestational hypertension, gestational diabetes mellitus, biliary stones and fatty liver, were all recorded.
2) Delivery: These data included methods of delivery (including vaginal delivery, cesarean section and forceps-assisted delivery), gender of the newborn, weight of the newborn, whether the newborn was macrosomic, gestational age of delivery, whether the newborn was premature, whether the newborn was asphyxia, whether the newborn was complicated with HTG-AP, whether the newborn was transferred to the neonatal intensive care unit (NICU) and whether the pregnant woman was transferred to the intensive care unit (ICU).
3) Laboratory examination before delivery: These data included TG, cholesterol, low-density lipoprotein (LDL), high-density lipoprotein (HDL), apolipoprotein (APO) A1, APO B, alanine aminotransferase (ALT), aspartate aminotransferase (AST), gamma glutamyl transpeptidase (γ-GGTP), total bilirubin (TBIL), direct bilirubin (DBIL), albumin, urea nitrogen (BUN), creatinine, urinary inhibition, potassium, sodium, calcium, hypocalcaemia, white blood cells, platelets, absolute neutrophil count (ANC), absolute lymphocyte count (ALC), ANC/ALC (N/L) ratio, hypersensitive C-reactive protein (hs-CRP) and plasma D-dimer.
4) Blood purification treatment: These data included haemoperfusion, plasma exchange and continuous renal replacement therapy (CRRT).

All data were merged into a complete database by hospitalization numbers.

### Quality control

One obstetrician and one ICU doctor were selected for standardized guidance and training. Information on obstetrician patients (including pregnant women transferred to the ICU) with TGs ≥ 11.30 mmol/L was screened through the clinical laboratory system. The electronic medical record system was matched according to name and hospitalization number, and basic information about pregnant women and related treatment information were checked in electronic medical records. Those eligible for inclusion had to be registered with complete medical records.

### Statistical analysis

SPSS 25.0 software was used for statistical analysis. Measurement data conforming to a normal distribution were described by (*x* ± s), and comparison between groups was performed by *t*-test (or corrected *t*-test). Measurement data that did not conform to a normal distribution were described by M (*P*_25_, *P*_75_), and the rank-sum test was used for comparisons between groups. Statistical data are expressed as the number of cases and percentage. The χ^2^ test (or Fisher's exact probability method) was used for intergroup comparisons, and the trend test was used for intergroup comparisons of grade variables. *P* < 0.05 was considered statistically significant.

## Results

### Comparison of the general data of puerperas

To evaluate the effects of insulin treatment, we compared the general maternal data. We observed that the insulin treatment group had patients with higher BMIs before pregnancy (Z = −2.281, *P* = 0.023) and a higher proportion of pregnant women with diabetes (χ^2^ = 20.618, *P* < 0.001) and fatty liver (χ^2^ = 4.333, *P* = 0.037) than the control group. There were no significant differences in age, primiparity, hypertension during pregnancy and presence of biliary calculi between the treatment and control groups. However, the insulin treatment group achieved a greater gestational weight gain compliance rate (χ^2^ = 4.061, *P* = 0.044) than the control group (Table 1). These results suggested that insulin treatment could improve the gestational weight gain compliance rate.

**Table 1:** Comparison of general data of puerperas.

**Item**	**Control group (*n* = 365)**	**Insulin treatment group (*n* = 48)**	**χ^2^/Z– value**	***P*–value**
Age (*P_25_, P_75_*)	31 (28, 34)	31 (29, 34)	−0.050	0.960
Progestational BMI (*P_25_, P_75_*)	21.9 (20.7, 23.6)	22.3 (21.6, 24.6)	−2.281	0.023
Pregnant women with weight up to standard rate, %	74.2	87.5	4.061	0.044
Rate of primiparity, %	58.9	54.2	0.392	0.531
Rate of hypertension during pregnancy, %	22.5	20.8	0.065	0.798
Gestational diabetes			20.618^a^	< 0.001^a^
Rate of pregnant women without diabetes, %	65.8	39.6		
Rate of pregnant women with diet–controlled diabetes, %	30.7	41.7		
Rate of pregnant women with insulin–treated diabetes, %	3.6	18.8		
Rate of pregnant women with biliary calculi, %	0.3	2.1	0.350	0.554
Rate of pregnant women with fatty liver, %	0.3	4.2	4.333	0.037

^a^Trend test the unmarked: rank-sum test or chi-square test. The measurement data conforming to the normal distribution were described by x ± s, the measurement data not conforming to the normal distribution were described by M (P_25_, P_75_), and the counting data were presented by percentage.

### Comparison of laboratory examination before delivery

Laboratory examination and the percentage of blood purification treatments before delivery were compared between the insulin treatment and control groups. Patients in the insulin treatment group had lower prenatal TG (Z = −10.392, *P* < 0.001), cholesterol (Z = −8.494, *P* < 0.001), LDL (Z = −3.918, *P* < 0.001), APO A1 (t = 2.410, *P* = 0.019), cystatin (Z = −4.195, *P* < 0.001), incidence of hypocalcaemia (*P* = 0.036), and ALC (Z = −3.426, *P* = 0.001) and higher DBIL (Z = −2.338, *P* = 0.019), N/L ratio (Z = −2.751, *P* = 0.006), and Ca in blood (Z = −2.944, *P* = 0.003) (Table 2). There were no significant differences between the treatment and control groups for other laboratory examinations, including HDL, APO B, TBIL, albumin, blood BUN, creatinine, K, Na, white blood cells, platelets, ANC, hs-CRP, D–dimer contents and ALT, AST and γ-GGT activities in blood. The percentages of all blood purification treatments (blood perfusion, plasma exchange and CRRT) were also not significantly different between the two groups. These results indicated that insulin was effective in the treatment of severe hypertriglyceridaemia in the third trimester of pregnancy.

**Table 2:** Comparison of laboratory examination and blood purification treatment before delivery.

**Item**	**Control group (*n* = 365)**	**Insulin treatment group (*n* = 48)**	**χ^2^/Z–value**	***P*–value**
TG (*P_25_, P_75_*)	13.47(12.06, 15.85)	8.67 (8.37, 10.52)	−10.392	< 0.001
Cholesterol (*P_25_, P_75_*)	8.25 (7.29, 9.63)	5.72 (4.69, 6.61)	−8.494	< 0.001
LDL (*P_25_, P_75_*)	3.26 (2.48, 3.81)	2.23 (1.22, 3.23)	−3.918	< 0.001
HDL (*P_25_, P_75_*)	1.37 (1.16, 1.56)	1.40 (1.06, 1.81)	−1.048	0.295
APO A1	2.32 ± 0.38	2.13 ± 0.53	2.410^c^	0.019^c^
Apo B	1.22 ± 0.28	1.20 ± 0.44	0.363^c^	0.718^c^
ALT (*P_25_, P_75_*)	9.0 (7.0, 12.0)	10.0 (7.0, 14.7)	−1.067	0.286
AST (*P_25_, P_75_*)	17.0 (14.4, 20.0)	17.6 (15.0, 22.0)	−0.963	0.335
γ-GGT (*P_25_, P_75_*)	12.0 (8.4, 18.0)	15.7 (8.0, 20.5)	−1.112	0.266
TBIL (*P_25_, P_75_*)	7.0 (5.4, 8.7)	7.2 (5.3, 10.5)	−0.995	0.320
DBIL (*P_25_, P_75_*)	1.1 (0.7, 1.9)	1.4 (0.8, 2.6)	−2.338	0.019
Albumin (*P_25_, P_75_*)	35.3 (33.5, 37.1)	34.9 (33.8, 36.0)	−1.137	0.255
BUN (*P_25_, P_75_*)	3.69 (3.09, 4.40)	3.76 (2.93, 4.86)	−0.700	0.484
Creatinine (*P_25_, P_75_*)	44.7 (38.3, 50.0)	45.5 (38.9, 56.8)	−1.036	0.300
Cystatin (*P_25_, P_75_*)	1.25 (1.08, 1.51)	1.10 (0.91, 1.24)	−4.195	< 0.001
K (*P_25_, P_75_*)	3.97 (3.79, 4.16)	4.05 (3.85, 4.23)	−1.435	0.151
Na (*P_25_, P_75_*)	135.0 (133.4, 136.0)	134.9 (132.8, 135.9)	−1.450	0.147
Ca (*P_25_, P_75_*)	2.27 (2.20, 2.34)	2.30 (2.24, 2.39)	−2.944	0.003
Rate of Hypocalcaemia	8.2	0.0	—	0.036^b^
Leukocytes (*P_25_, P_75_*)	8.59 (7.31, 10.15)	8.68 (7.48, 10.42)	−0.496	0.620
Platelets (*P_25_, P_75_*)	196 (158, 232)	186 (153, 216)	−1.097	0.273
ANC (*P_25_, P_75_*)	6.40 (5.29, 7.75)	6.64 (5.23, 7.94)	−0.594	0.552
ALC (*P_25_, P_75_*)	1.51 (1.27, 1.91)	1.26 (1.03, 1.72)	−3.426	0.001
N/L (*P_25_, P_75_*)	4.07 (3.16, 5.28)	5.17 (3.24, 9.33)	−2.751	0.006
hs–CRP (*P_25_, P_75_*)	2.76 (1.49, 4.68)	2.92 (1.37, 4.66)	−0.250	0.802
Plasma D–dimer (*P_25_, P_75_*)	1.80 (1.35, 2.59)	1.83 (1.31, 2.32)	−0.418	0.676
Rate of pregnant women receiving blood perfusion, %	0.5	2.1	0.075^a^	0.784^a^
Rate of pregnant women receiving plasma exchange, %	0.8	0.0	—	1.00^b^
Rate of pregnant women receiving haemofiltration, %	0.5	0.000	—	1.00^b^

TG, triglyceride; LDL, low–density lipoprotein; HDL, high–density lipoprotein; APO, apolipoprotein; ALT, alanine aminotransferase; AST, aspartate aminotransferase; γ-GGT, gamma glutamyl transferase; TBIL, total bilirubin; DBIL, direct bilirubin; BUN, urea nitrogen; ANC, absolute neutrophil count; ALC, absolute lymphocyte count; N/L, ANC/ALC; hs–CRP, hypersensitive C–reactive protein.

^a^Corrected chi–square test, ^b^Fisher's exact test, ^c^Corrected t–test, the unmarked: rank-sum test. The measurement data conforming to the normal distribution were described by x ± s, the measurement data not conforming to the normal distribution were described by M (P_25_, P_75_), and the counting data were presented by percentage.

### Comparison of delivery outcomes

Delivery outcomes were compared between the insulin treatment group and the control group. We found earlier gestational age (Z = −3.427, *P* = 0.001), lower neonatal weight (Z = −2.200, *P* = 0.028), lower incidence of macrocephaly (χ^2^ = 4.092, *P* = 0.043) and lower incidence of maternal referral to ICU treatment (*P* = 0.014) in the insulin treatment group than in the control group (Table 3). There were no significant differences in delivery mode, gender of newborn, neonatal asphyxia rate, incidence of premature delivery, HTG-AP or transfer to NICU treatment between the two groups. These results revealed that insulin treatment improved the delivery outcomes of severe hypertriglyceridaemia in the third trimester of pregnancy.

**Table 3:** Comparison of delivery between insulin treatment group and control group.

**Item**	**Control group (*n* = 365)**	**Insulin treatment group (*n* = 48)**	**χ^2^/Z–value**	***P*–value**
Delivery way			0.818	0.624
Rate of eutocia, %	32.6	27.1		
Rate of cesarean delivery, %	65.8	72.9		
Rate of forceps delivery, %	1.6	0.0		
Rate of delivery boy, %	52.1	60.4	1.191	0.275
Neonatal weight (*P_25_, P_75_*)	3400 (3093, 3700)	3200 (2805, 3605)	−2.200	0.028
Rate of macrosomia, %	16.4	4.2	4.092^a^	0.043^a^
Rate of neonatal asphyxia, %	1.4	4.2	0.667^a^	0.414^a^
Delivery gestational age (*P_25_, P_75_*)	39.0 (38.1, 39.4)	38.1 (37.2, 39.1)	−3.427	0.001
Percentage			0.733	1.000
Rate of mature birth, %	89.9	91.7		
Rate of premature birth, %	9.9	8.3		
Rate of stillbirth, %	0.3	0.0		
Rate of HTG–AP, %	2.7	0.0	—	0.614^b^
Rate of pregnant women shifted to NICU, %	7.7	8.3	0.000	1.000
Rate of pregnant women shifted to ICU, %	10.4	0.000	—	0.014^b^

HTG–AP, hypertriglyceridaemia acute pancreatitis; NICU: neonatal intensive care unit; ICU, intensive care unit.

^a^Corrected chi–square test, ^b^Fisher's exact test, the unmarked: rank-sum test or chi-square test. The measurement data conforming to the normal distribution were described by x ± s, the measurement data not conforming to the normal distribution were described by M (P_25_, P_75_), and the counting data were presented by percentage.

## Discussion

The low incidence of severe HTG restricts the study of the risk factors for this disease. Therefore, there is still a lack of clear guidance for the prevention of severe HTG. HTG is mostly caused by gene mutation or familial HTG and non-hereditary and non-familial factors, such as pregnancy, obesity, alcohol dependence, uncontrolled diabetes, hypothyroidism, chronic renal failure and drug usage (namely, estrogen drugs, glucocorticoids, immunosuppressants and antipsychotics) (11). In this study, we found that a higher BMI before pregnancy and a higher proportion of pregnant women with diabetes and fatty liver were associated with earlier occurrence of severe HTG in late pregnancy. In addition, less severe HTG patients (5.65–11.30 mmol/L) at 30–32 weeks of gestation who developed severe HTG before delivery, even with low-fat diet treatment, had a higher progestational BMI, lower gestational weight gain compliance rate, higher TG and cholesterol before delivery, higher rates of macrosomia, HTG-AP and pregnant women shifted to the NICU during delivery than non-progressive HTG patients (Supplementary Table S1). Thus, overweight or obesity before pregnancy could be a risk factor for severe HTG, and weight control during pregnancy could improve pregnancy outcomes. Pregnant women with elevated TGs in early pregnancy had an increased risk of gestational diabetes and macrosomia (12). Therefore, for intervention with a low-fat diet and exercise, the optimal time to screen for dyslipidaemia is prepregnancy or early pregnancy (13). For late pregnancy, proper weight gain during pregnancy, management of pregnancy complications (diabetes, hypothyroidism, etc.) and intensive insulin treatment should also be emphasized.

Approximately 15–20% of severe HTG patients develop HTG-AP (7), and up to 50% of acute pancreatitis in pregnancy is associated with HTG (14). HTG is the second major cause of acute pancreatitis in China, and the trend is that it occurs in younger pregnant women with more severe disease (15). Acute pancreatitis in pregnancy (APIP) has led to maternal and fetal mortality rates of 37 and 60%, respectively, in the past (4). With the improvement of timely identification and treatment, the mortality rate has decreased to 1 and 20%, respectively, which is better but still threatens the health of mothers and children (2). Consistent with a previous study (4), the incidence of HTG-AP in this study was 0.8/10000 (10/118622), all of which occurred in the control group in contrast with none in the insulin treatment group, suggesting an effect of insulin in the third trimester on preventing the occurrence of HTG-AP. Therefore, in midwifery institutions, it is recommended that blood lipid levels be monitored every 1–2 weeks for pregnant women with TG > 5.65 mmol/L in the third trimester of pregnancy and intensive insulin therapy be initiated in a timely manner when TG > 11.30 mmol/L to reduce the occurrence of HTG-AP and avoid adverse pregnancy outcomes.

There were some limitations in this study. The timing of severe HTG occurrence in the treatment and control groups was not the same; this difference might introduce bias but not alter the conclusion. This treatment group in this study included patients with severe HTG (TG ≥ 11.30 mmol/L) that occurred at 30–32 weeks of gestation who were treated with a low-fat diet and insulin. The best control group would have been severe HTG patients at the same stage who were treated with a low-fat diet. However, the incidence of severe HTG in pregnancy from our hospital was 0.348% (413/11862). Among them, only 48/413 severe HTG cases occurred at 30–32 weeks of gestation. The remaining severe HTG (365/413) patients had TGs of 5.65–11.30 mmol/L at 30–32 weeks of gestation, received low-fat diet treatment, but still developed severe HTG before delivery (at 37–41 weeks of pregnancy). Their conditions were not as severe as in the treatment group due to delayed disease occurrence as well as lower TG and BMI before pregnancy and the proportion of pregnant women with diabetes and fatty liver at 30–32 weeks of gestation. However, patients in the insulin treatment group achieved even better clinical outcomes, including higher gestational weight gain compliance rates, lower TG levels before delivery and better delivery outcomes than those in the control group. Therefore, we concluded that insulin was effective in treating severe HTG in the third trimester of pregnancy. In the future, a study of large samples from multiple centers can be designed to analyse the risk factors for severe HTG more comprehensively and dynamically and to determine the effects of insulin on HTG at the same level of severity. Moreover, the standardized management model for severe HTG during pregnancy should be adopted to reduce bias in the study as well as to prevent HTG-AP occurrence.

## Data availability statement

The original contributions presented in the study are included in the article/supplementary material, further inquiries can be directed to the corresponding author.

## Ethics statement

The studies involving human participants were reviewed and approved by the Ethics Committee of the Maternal and Child Health Hospital of Hubei Province. The patients/participants provided their written informed consent to participate in this study.

## Author contributions

DZ: research design, implementation, data collection, analysis, and paper writing. GS and JH: participated in research design and guidance. QG: participated in research design, implementation, supervision of data collection and analysis, paper revision, and financial support. All authors contributed to the article and approved the submitted version.

## Funding

This study was funded by Wuhan Young and Middle-aged Medical Backbone Talent Training Project (the eighth batch), the Research Foundation of the Maternal and Child Health Hospital of Hubei Province (No. 2021SFYM008).

## Conflict of interest

The authors declare that the research was conducted in the absence of any commercial or financial relationships that could be construed as a potential conflict of interest.

## Publisher's note

All claims expressed in this article are solely those of the authors and do not necessarily represent those of their affiliated organizations, or those of the publisher, the editors and the reviewers. Any product that may be evaluated in this article, or claim that may be made by its manufacturer, is not guaranteed or endorsed by the publisher.
