# Supplementary material for: Efficacy of insulin in treating severe hypertriglyceridaemia in the third trimester of pregnancy
Source: Front Med (Lausanne). 2022 Nov 2;9:977620. doi: 10.3389/fmed.2022.977620 (PMC9666498; doi:10.3389/fmed.2022.977620)
Supplement: Supplementary file 1 [file Data_Sheet_1.docx]

Supplementary Figure 1. Flow diagram of the study showing the insulin treatment group and the control group for severe HTG.

2017.1.1~2021.9.30 The women who received clinical examination and delivered in Hubei Maternal and Child Health Hospital (n=118622)

Inclusion criteria:

18-45 years old;

Delivery of a single live birth;

HTG in the third trimester;

Compliance with medical advice for treatment with low-fat diet

Insulin treatment group

treated with low-fat diet and insulin

treated with low-fat diet

TG>5.65 mmol/L

30-32 weeks of gestation

Severe HTG (TG≥11.30 mmol/L, n=48)

Less severe HTG (TG 5.65~11.30 mmol/L, n=7056)

before delivery

Severe HTG (TG≥11.30 mmol/L, n=365)

Less severe HTG (TG 5.65~11.30 mmol/L, n=6691)

Control group

Nonprogressive HTG (n=400; Randomly select)

Exclusion criteria:

Incomplete medical records;

Noncompliance with medical advice for treatment

Supplementary Table 1. Comparison of general data of puerperas, laboratory examination before delivery, and delivery between control group (severe TG) and TG group.

|  | General data of puerperas | | | |
| --- | --- | --- | --- | --- |
|  | Control group (severe HTG, n=365) | HTG group (n=400) | c2/Z value | P value |
| Age (P25, P75) | 31 (28, 34) | 31 (28, 34) | -0.456 | 0.648 |
| Progestational BMI (P25, P75) | 21.9 (20.7, 23.6) | 21.5(19.6,23.5) | -3.733 | <0.001 |
| Pregnant women with weight up to standard rate, % | 74.2 | 81.8 | 6.297 | 0.012 |
| Rate of primiparity, % | 58.9 | 61.5 | 0.537 | 0.464 |
| Rate of hypertension during pregnancy, % | 22.5 | 18.0 | 2.367 | 0.124 |
| Gestational diabetes |  |  | 0.686 | 0.710 |
| Rate of pregnant women without diabetes, % | 65.8 | 68.5 |  |  |
| Rate of pregnant women with diet-controlled diabetes, % | 30.7 | 28.0 |  |  |
| Rate of pregnant women with insulin-treated diabetes, % | 3.6 | 3.5 |  |  |
| Rate of pregnant women with biliary calculi, % | 0.3 | 0.8 | 0.168b | 0.682b |
| Rate of pregnant women with fatty liver, % | 0.3 | 0.0 | — | 0.477 |
|  | Laboratory examination before delivery | | | |
| TG (*P25*, *P75*) | 13.47 (12.06, 15.85) | 6.71(6.09,7.75) | -23.912 | <0.001 |
| Cholesterol (*P25*, *P75*) | 8.25 (7.29, 9.63) | 6.58(5.81,7.63) | -14.026 | <0.001 |
| LDL (*P25*, *P75*) | 3.26 (2.48, 3.81) | 3.65(2.95,4.36) | -6.568 | <0.001 |
| HDL (*P25*, *P75*) | 1.37 (1.16, 1.56) | 1.61(1.44,1.80) | -11.470 | <0.001 |
| APO A1 | 2.32(2.05,2.56) | 2.42(2.19,2.67) | -3.825 | <0.001 |
| Apo B | 1.21(1.04,1.40) | 1.20(1.03,1.42) | -0.274 | 0.784 |
| ALT (*P25*, *P75*) | 9.0 (7.0, 12.0) | 9.0 (7.0, 12.0) | -0.192 | 0.848 |
| AST (*P25*, *P75*) | 17.0 (14.4, 20.0) | 18.0(15.0,21.0) | -2.596 | 0.009 |
| γ-GGT (*P25*, *P75*) | 12.0 (8.4, 18.0) | 11.0(8.0,16.0) | -2.777 | 0.005 |
| TBIL (*P25*, *P75*) | 7.0 (5.4, 8.7) | 6.5(5.1,8.4) | -1.867 | 0.062 |
| DBIL (*P25*, *P75*) | 1.1 (0.7, 1.9) | 1.3(0.7,2.1) | -1.438 | 0.151 |
| Albumin (*P25*, *P75*) | 35.3 (33.5, 37.1) | 35.3(33.9,37.2) | -0.944 | 0.345 |
| BUN (*P25*, *P75*) | 3.69 (3.09, 4.40) | 3.70(3.14,4.41) | -0.639 | 0.523 |
| Creatinine (*P25*, *P75*) | 44.7 (38.3, 50.0) | 49.1(44.5,56.2) | -8.929 | <0.001 |
| Cystatin (*P25*, *P75*) | 1.25 (1.08, 1.51) | 1.19(1.03,1.40) | -2.980 | 0.003 |
| K (*P25*, *P75*) | 3.97 (3.79, 4.16) | 4.09(3.93,4.28) | -6.853 | <0.001 |
| Na (*P25*, *P75*) | 135.0 (133.4, 136.0) | 136.0(135.0,137.0) | -7.064 | <0.001 |
| Ca (*P25*, *P75*) | 2.27 (2.20, 2.34) | 2.28(2.21,2.35) | -2.303 | 0.021 |
| Rate of Hypocalcaemia (%) | 8.2 | 1.3 | 21.232 | <0.001 |
| Leukocyte (*P25*, *P75*) | 8.59 (7.31, 10.15) | 8.61(7.17,10.09) | -0.654 | 0.513 |
| Platelet (*P25*, *P75*) | 196 (158, 232) | 198(168,232) | -1.413 | 0.158 |
| ANC(*P25*, *P75*) | 6.40 (5.29, 7.75) | 6.52(5.28,7.78) | -0.025 | 0.980 |
| ALC (*P25*, *P75*) | 1.51 (1.27, 1.91) | 1.44(1.23,1.77) | -2.506 | 0.012 |
| N/L (*P25*, *P75*) | 4.07 (3.16, 5.28) | 4.38(3.40,5.36) | -1.638 | 0.101 |
| hs-CRP (*P25*, *P75*) | 2.76 (1.49, 4.68) | 2.80(1.73,4.61) | -0.836 | 0.403 |
| Plasma d-dimer (*P25*, *P75*) | 1.80 (1.35, 2.59) | 1.67(1.26,2.17) | -2.953 | 0.003 |
| Rate of pregnant women receiving blood perfusion, % | 0.5 | 0.0 | — | 0.227c |
| Rate of pregnant women receiving plasma exchange, % | 0.8 | 0.0 | — | 0.108c |
| Rate of pregnant women receiving haemofiltration, % | 0.5 | 0.0 | — | 0.227c |
|  | delivery | | | |
| Delivery way |  |  | 3.078 | 0.215 |
| Rate of eutocia, % | 32.6 | 37.5 |  |  |
| Rate of caesarean delivery, % | 65.8 | 61.8 |  |  |
| Rate of forceps delivery, % | 1.6 | 0.8 |  |  |
| Rate of delivery boy, % | 52.1 | 55.0 | 0.666 | 0.415 |
| Neonatal weight (P25, P75) | 3400 (3093, 3700） | 3350(3015,3638) | -1.228 | 0.22 |
| Rate of macrosomia, % | 16.4 | 7.8 | 13.746 | <0.001 |
| Rate of neonatal asphyxia, % | 1.4 | 0.3 | 1.805b | 0.179b |
| Delivery gestational age (P25, P75) | 39.0 (38.1, 39.4） | 39.0(38.1,39.4) | -0.274 | 0.784 |
| Percentage |  |  | 1.662c | 0.412c |
| Rate of mature birth, % | 89.9 | 91.8 |  |  |
| Rate of premature birth, % | 9.9 | 8.3 |  |  |
| Rate of stillbirth, % | 0.3 | 0.0 |  |  |
| Rate of HTG-AP, % | 2.7 | 0.0 | — | 0.001 |
| Rate of pregnant women shifted to NICU, % | 7.7 | 6.3 | 0.598 | 0.439 |
| Rate of pregnant women shifted to ICU, % | 10.4 | 1.3 | 30.192 | <0.001 |

aTrend test, bCorrected chi-square test, cFisher's exact test, dCorrected t test, the unmarked: the rank-sum test or chi-square test. The measurement data conforming to the normal distribution were described by x ± s, the measurement data not conforming to the normal distribution were described by M (*P25*, *P75*), and the counting data were presented by percentage.
